# Floral Evolution of *Philodendron* Subgenus *Meconostigma* (Araceae)

**DOI:** 10.1371/journal.pone.0089701

**Published:** 2014-02-26

**Authors:** Letícia Loss de Oliveira, Luana Silva Braucks Calazans, Érica Barroso de Morais, Simon Joseph Mayo, Carlos Guerra Schrago, Cassia Mônica Sakuragui

**Affiliations:** 1 Departamento de Genética, Instituto de Biologia, Universidade Federal do Rio de Janeiro, Rio de Janeiro, Rio de Janeiro, Brazil; 2 Departamento de Botânica, Instituto de Biologia, Universidade Federal do Rio de Janeiro, Rio de Janeiro, Rio de Janeiro, Brazil; 3 Departamento de Botânica, Museu Nacional, Universidade Federal do Rio de Janeiro, Rio de Janeiro, Rio de Janeiro, Brazil; 4 Herbarium, Royal Botanic Gardens, Kew, Richmond, Surrey, United Kingdom; Universidade Federal do Rio de Janeiro, Brazil

## Abstract

Elucidating the evolutionary patterns of flower and inflorescence structure is pivotal to understanding the phylogenetic relationships of Angiosperms as a whole. The inflorescence morphology and anatomy of *Philodendron* subgenus *Meconostigma*, belonging to the monocot family Araceae, has been widely studied but the evolutionary relationships of subgenus *Meconostigma* and the evolution of its flower characters have hitherto remained unclear. This study examines gynoecium evolution in subgenus *Meconostigma* in the context of an estimated molecular phylogeny for all extant species of subgenus *Meconostigma* and analysis of ancestral character reconstructions of some gynoecial structures. The phylogenetic reconstructions of all extant *Meconostigma* species were conducted under a maximum likelihood approach based on the sequences of two chloroplast (*trn*k and *matK*) and two nuclear (ETS and 18S) markers. This topology was used to reconstruct the ancestral states of seven floral characters and to elucidate their evolutionary pattern in the *Meconostigma* lineage. Our phylogeny shows that *Meconostigma* is composed of two major clades, one comprising two Amazonian species and the other all the species from the Atlantic Forest and Cerrado biomes with one Amazonian species. The common ancestor of the species of subgenus *Meconostigma* probably possessed short stylar lobes, long stylar canals, a stylar body, a vascular plexus in the gynoecium and druses in the stylar parenchyma but it is uncertain whether raphide inclusions were present in the parenchyma. The ancestral lineage also probably possessed up to 10 ovary locules. The evolution of these characters seems to have occurred independently in some lineages. We propose that the morphological and anatomical diversity observed in the gynoecial structures of subgenus *Meconostigma* is the result of an ongoing process of fusion of floral structures leading to a reduction of energy wastage and increase in stigmatic surface.

## Introduction

The highly diverse monocot family Araceae Juss. comprises 3,319 currently recognized species classified into 118 genera [1]. The Neotropical genus *Philodendron* Schott is of special interest as it represents the second most diverse genera of the family, with 489 accepted species [2], and is one of the most conspicuous and well-known groups of epiphytic and hemiepiphytic plants [3]. The current taxonomy of *Philodendron* follows Mayo [4], Grayum [5], Croat [6] and Sakuragui *et al.* [7] and comprises three subgenera: *Philodendron*, *Pteromischum* and *Meconostigma*, the latter with 21 species.

*Philodendron* is composed of very showy and horticulturally durable plants. These features are especially notable in subgenus *Meconostigma* and as a consequence, species of this subgenus are widely used in horticulture as valuable ornamental plants [8]. The roots of some species, such as *P. corcovadense* [9] and *P. williamsii* [10] are used by traditional Brazilian populations for making rustic craft products that are widely sold in urban centers.

**Figure 1:**
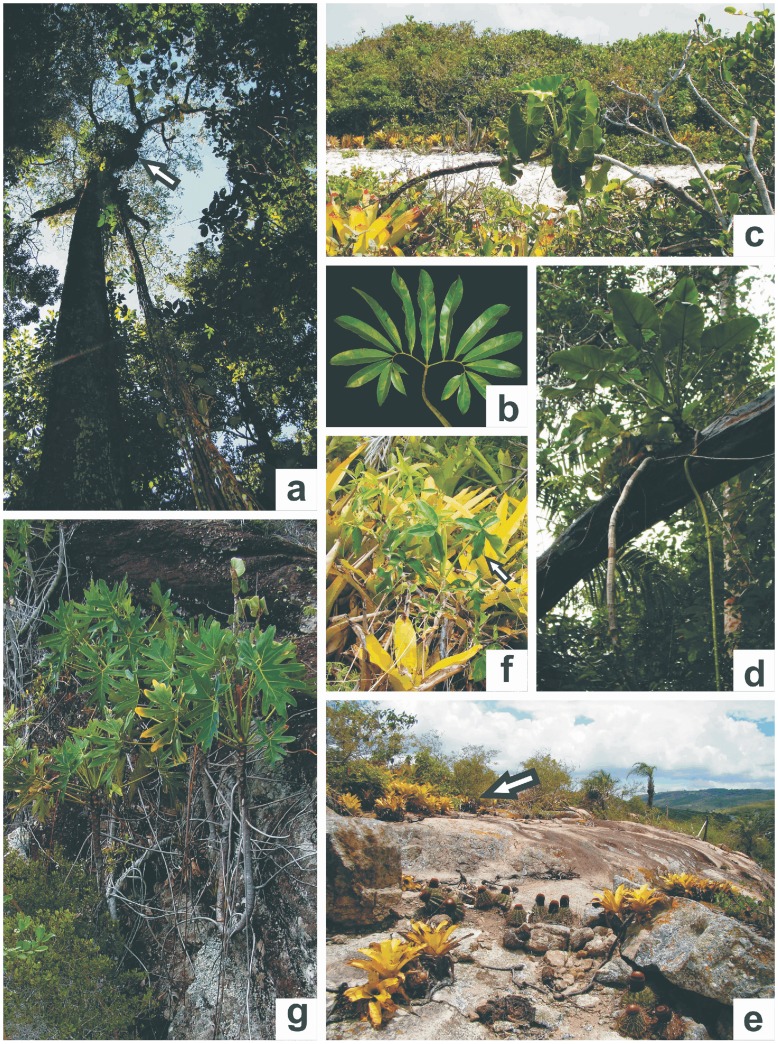
Specimens of *Philodendron* subg. Meconostigma. a. A hemiepiphytic specimen of *P. goeldii* indicated by the white arrow in the Amazon Forest (Manaus city, Amazonas state), b. *P. goeldii* leaf. c. A P. corcovadense individual growing directly on sand in the Atlantic Rainforest, Restinga vegetation (Maricá city, Rio de Janeiro state), d. A hemiepiphytic specimen of *P. williamsii* in the Atlantic Rainforest *s.s.*, (Itacaré city, Bahia state), e. A population of *P. leal-costae* pointed by the white arrow in the Caatinga inselberg (Milagres city, Bahia state), f. Bromelicolous habit of *P. leal-costae*, g. *P. saxicola* rupicolous habit in the Cerrado biome, *campo rupestre* vegetation (Lençóis city, Bahia state).

The three subgenera of *Philodendron* were recovered as monophyletic by the molecular study of Gauthier *et al.* [9]. Subgenus *Meconostigma* has been recognized since the first taxonomic proposal of *Philodendron* by Schott in 1829 and was most recently revised by Mayo [8] with later updates by Gonçalves and Salviani [12]. Members of this subgenus are well distinguished by diagnostic morphological characters as well as by a conspicuous geographical distribution [8], [12], ranging from the Amazonian and Atlantic forests to savanna-like landscapes (Cerrado biome), as displayed in Figure 1.

Although the monophyly of subgenus *Meconostigma* is indicated by morphological, anatomical [13], [4], [8] and molecular analyses [11], the detailed phylogenetic relationships of its 21 extant species remained to be established. Gauthier *et al.* [11], who analyzed 6 species, made the largest molecular survey of subgenus *Meconostigma* hitherto.

Due to their high level of variability, flowers have been one of the main sources of characters used to investigate the phylogeny of *Philodendron* species [14]. Each female flower in *Philodendron* consists of a single gynoecium lacking staminodes or perianth parts. Together, they form a well-defined female zone at the base of the spadix. An important characteristic of the gynoecium is the presence of a separate stylar canal for each carpel, a feature used by Mayo [4] to discuss the taxonomy and evolution of *P.* subgenus *Meconostigma*.

However, in this group, morphological characters present significant plasticity, which increases the frequency of homoplasies in phylogenetic analysis. Moreover, Mayo [13], [4] suggested that parallelism and convergence may be widespread in the evolution of subgenus *Meconostigma*. Such modes of evolution would hamper the phylogenetic reconstruction of this subgenus using morphology and anatomy alone.

In this study, we have inferred the phylogeny of extant *P*. subgenus *Meconostigma* species using both nuclear and plastid molecular markers. With the aim of understanding the evolutionary history of the inflorescence of subgenus *Meconostigma*, this new phylogeny was used to investigate the evolution of floral characters in the group, with a focus on the gynoecium.

Our findings suggest that the morphological diversity observed in the gynoecium of *Meconostigma* species is the result of an ongoing process of fusion of its flower structures leading to a reduction of energy wastage and increase in stigmatic surface and, as a consequence, the chances of fertilization.

## Materials and Methods

### Species and Gene Sampling

We generated molecular sequences for the nuclear 18S and external transcribed spacer (ETS) and the *trn*K intron and maturase K (*matK)* gene from the chloroplast of all extant species of subgenus *Meconostigma* and of *Philodendron* subgenus *Philodendron* (*P. pedatum*) and *Philodendron* subgenus *Pteromischum* (*P. oblongum*). We also included one species from *Homalomena* (*H. cochinchinensis*) as outgroup, the genus most closely related to *Philodendron*. Information on GenBank accession numbers and voucher of the species studied are listed in Table S1 and Table S2 in File S1, respectively.

### Ethics Statement

All living tissues were collected following the guidelines and jurisprudence of the Brazilian Ministry of Environment (MMA): SISBIO authorization no. 25755-1. Sítio Roberto Burle-Marx, Marcus Nadruz, Eduardo Gonçalves, Harri Lorenzi, Lourdes Soares and Leland Mayano provided live material from cultivated plants, which are free from MMA legislation.

### DNA Isolation, Amplification and Sequencing

Genomic DNA was isolated with QIAGEN DNeasy Blood & Tissue kit from silica-dried or fresh leaves. The amplification and sequencing were conducted using the primers listed in Table S3 in File S1. Sequencing reactions were performed in the Applied Biosystems 3730xl automatic sequencer. The consensus sequences were generated with Genious version 5.5.3 [15].

### Alignment and Phylogenetic Analysis

Sequences of the molecular markers were individually aligned in MUSCLE [16]. Alignments were manually adjusted in SeaView [17] and posteriorly concatenated into a single supermatrix of 3,323 base pairs. Phylogenetic inferences were performed using the maximum likelihood (ML) method as implemented in PhyML 3.0 [18] using the GTR+G model of sequence evolution with both concatenated and isolated sequences. Bayesian analysis relied on Mr. Bayes 3.2.2 [19], [20], using a random starting tree and four rate categories. The Markov chain Monte Carlo (MCMC) chains ran for 1.000.000 generations, with trees sampled every 100th generation and a burn-in of 1000 trees. Model choice was conducted in Modeltest, using the likelihood ratio test. Statistical confidence of clades was assessed by the approximate likelihood ratio test statistics - aLRT [21].

In order to evaluate the genetic similarities between species shown as very closely related in our ML and Bayesian phylogenies, we calculated their pairwise genetic distances with MEGA 5.2.2 [22] based on our concatenated matrix.

### Ancestral State Reconstruction of Gynoecium

**Figure 2:**
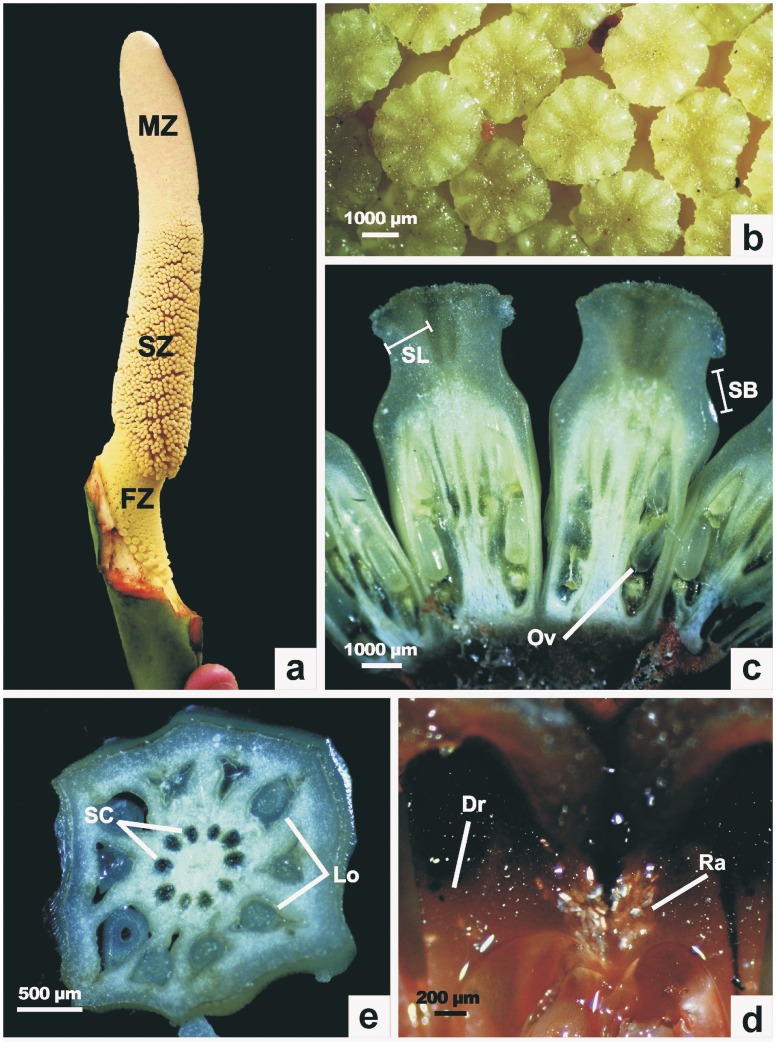
Morphological characters of *Philodendron* subg. *Meconostigma*. a. *P. bipinnatifidum* inflorescence as an example of a typical *Philodendron* subg. *Meconostigma* inflorescence, b. Upper view of some of the *P. bipinnatifidum* female flowers showing stylar lobes, c. Longitudinal cut of *P. bipinnatidifum* female flowers, d. Transversal cut of a *P. bipinnatifidum* female flower, e. Longitudinal cut of a *P. adamantinum* female flower. Acronyms list: MZ = male zone; SZ = sterile male zone; FZ = female zone; SL = stylar lobes; SB = stylar body; SC = stylar canals; Ov = ovules; Lo = locules; Dr = druses; Ra = raphides.

To infer evolutionary changes in the gynoecium, we composed a reduced data set containing the 22 species that were characterized morphologically. It consisted of 19 species of subgenus *Meconostigma* (*P. xanadu* and *P. leal-costae* are absent in this analysis) with two other *Philodendron* species {subgenus *Philodendron* (1), subgenus *Pteromischum* (1)} and one *Homalomena* species as outgroup. The morphological character states of the gynoecium are listed in Table S4 in File S1, as well as the state codes used to construct our matrix. Figure 2 illustrates these characters. The terminology used to name the characters follows the definitions of Mayo [4].

Ancestral character state reconstruction was performed in Mesquite version 2.73 [23] using the Markov k-state one-parameter model, which assumes a homogeneous probability of change between the number of states of a character. The transition parameters of the model were estimated from the phylogram obtained by the ML tree.

## Results

### Phylogenetic Relationships among *Meconostigma* Species

**Figure 3:**
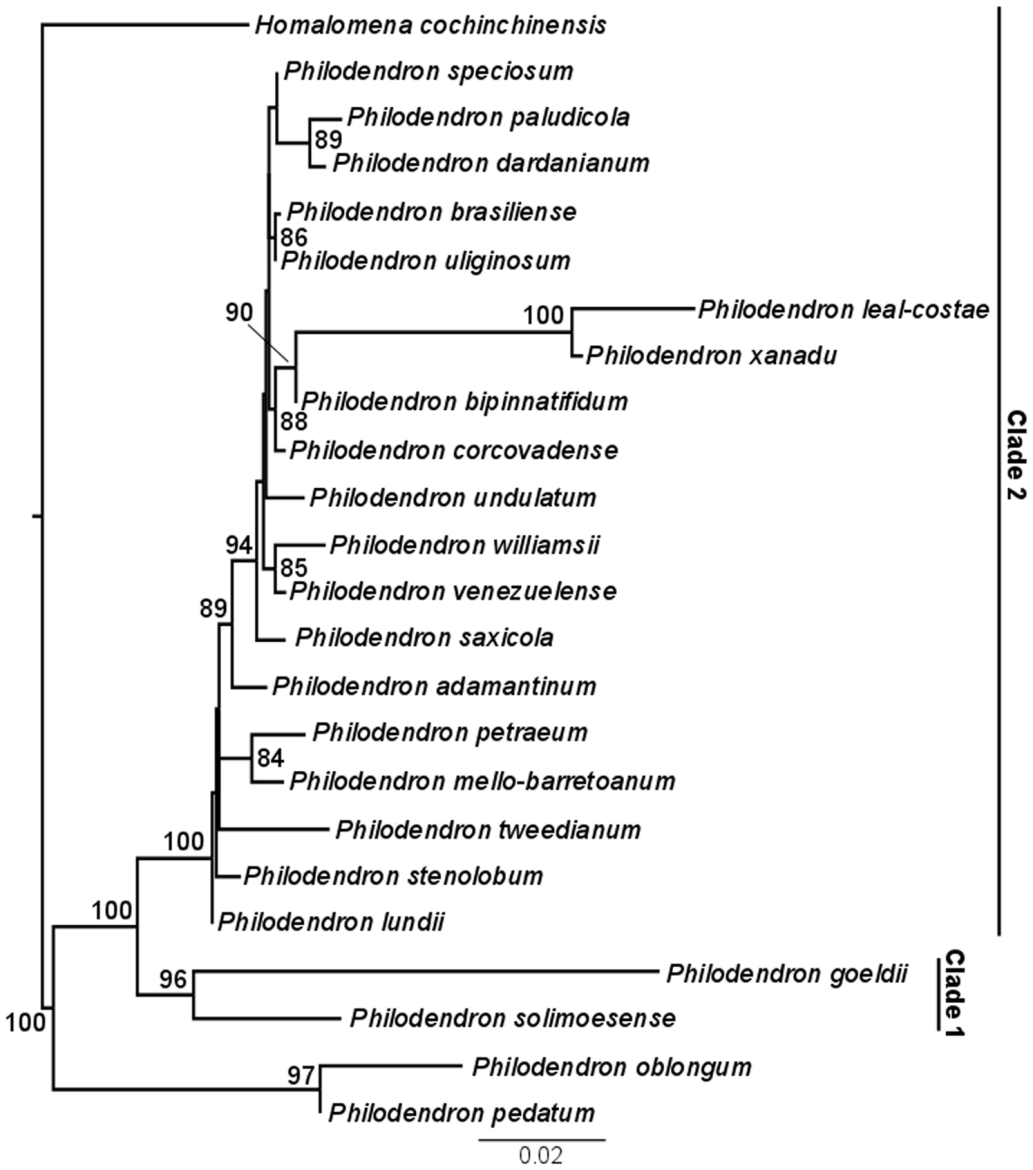
Maximum-likelihood phylogeny of *Meconostigma* species based on ETS and *matK* markers. aLRT values ≥85% are indicated in the branch nodes.

**Figure 4:**
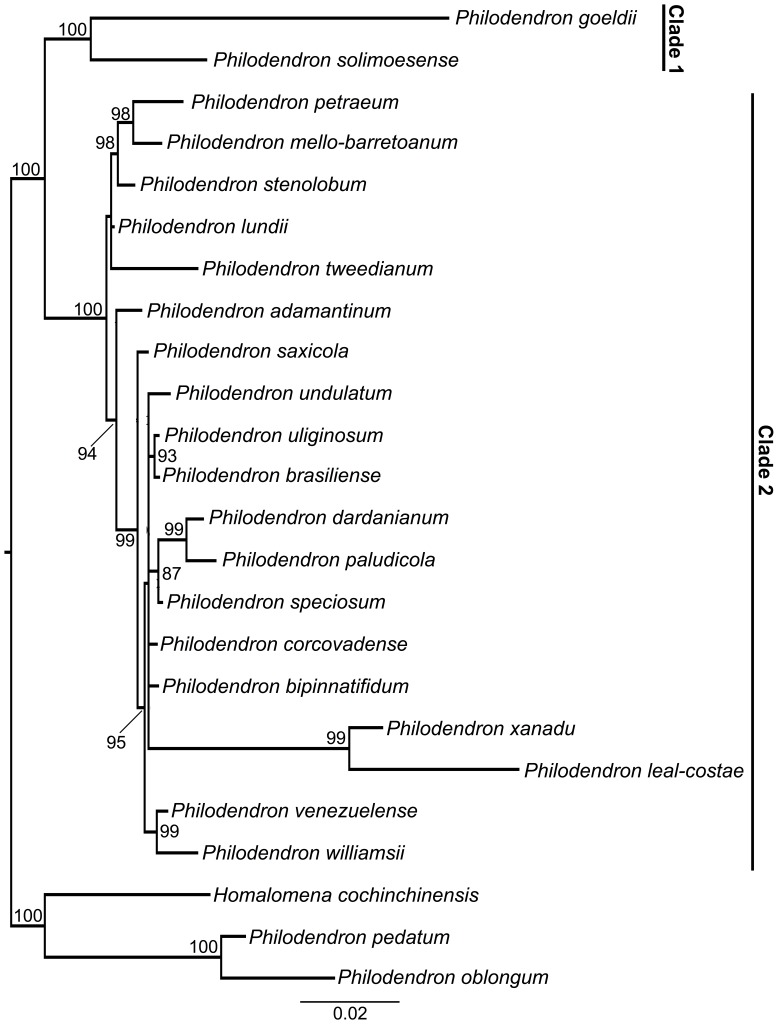
Bayesian analysis phylogeny of *Meconostigma* species based on ETS and *matK* markers. Posterior probabilities ≥85% are indicated in the branch nodes.

As displayed in Figure 3 and Figure 4, subgenus *Meconostigma* was recovered as monophyletic with 100% aLRT support and 100% posterior probability, respectively. The ML likelihood and Bayesian analysis provided very similar results. In general, the species relationships in the phylogeny reflect morphological similarities among them.

Within the subgenus, two major lineages could be identified in the ML and Bayesian phylogeny. One, hereafter referred to as Clade 1, consisted of two Amazonian species – *Philodendron solimoensense* and *Philodendron goeldii* (96% aLRT support, 100% posterior probability) and the other (Clade 2) consisting of a diverse assemblage of species from Amazonian and non-Amazonian biomes.

Clade 2 presented *P. lundii* as the basal-most species in the ML phylogeny (Figure 3). However, the phylogeny based on the Bayesian approach did not recover a single species as the basal-most among the others, instead, it presented two minor clades within Clade 2 (Figure 4), consisting of the only difference between the two trees.

*Philodendron venezuelense*, the remaining Amazonian species of the subgenus, was recovered as sister species of *P*. *williamsii*, an Atlantic forest species. Interestingly, the morphologically similar species *P. brasiliense* and *P. uliginosum* were recovered as sister groups in our phylogeny; both occur in damp to periodically flooded soils in open, *campo rupestre* vegetation. Unlike the other Clade 2 species, *P. leal-costae* and *P. xanadu* presented very long branch lengths in both ML and Bayesian analysis phylogenies (Figure 3, Figure 4), possibly due to their high evolutionary rate.

We calculated the genetic distance between the sister species that were most closely related on Clade 2. The results indicate low pairwise distances: *P. paludicola* and *P. dardanianum* (0.011); *P. brasiliense* and *P. uliginosum* (0.008); *P. williamsii* and *P. venezuelense* (0.008); *P. petraeum* and *P. mello-barretoanum* (0.011).

**Figure 5:**
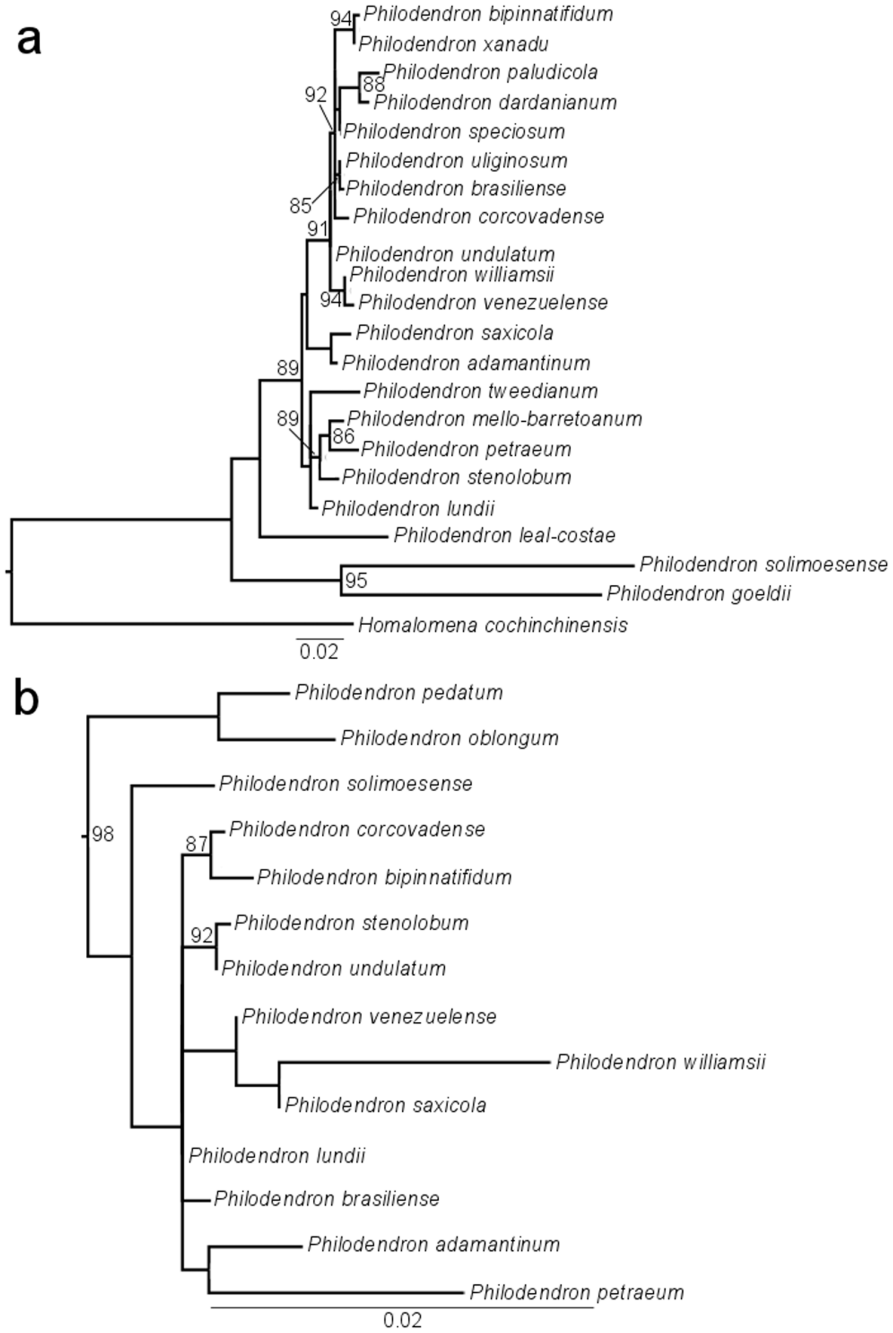
Maximum-likelihood phylogenies based on the isolated markers ETS and *matK*. a. Phylogeny based on ETS marker, b. Phylogeny based on *matK* marker. aLRT values ≥85% are indicated in the branch nodes.

In comparing the phylogenies based on the isolated markers (Figure 5), that based on 18S – ETS (Figure 5*a*) provided a better resolution for the identification of closely related species. On the other hand, *matK* – *trn*K marker (Figure 5*b*) provided a tree with very short branch lengths and more unresolved clades. Although these phylogenies did not comprise our complete taxon sampling, overall, the relationships among the species are similar to the ones recovered with the concatenated dataset, presenting *Meconostigma* as a monophyletic clade and Amazonian species as basal-most of the subgenus.

### Ancestral State Reconstruction of *Meconostigma* Gynoecium

**Figure 6:**
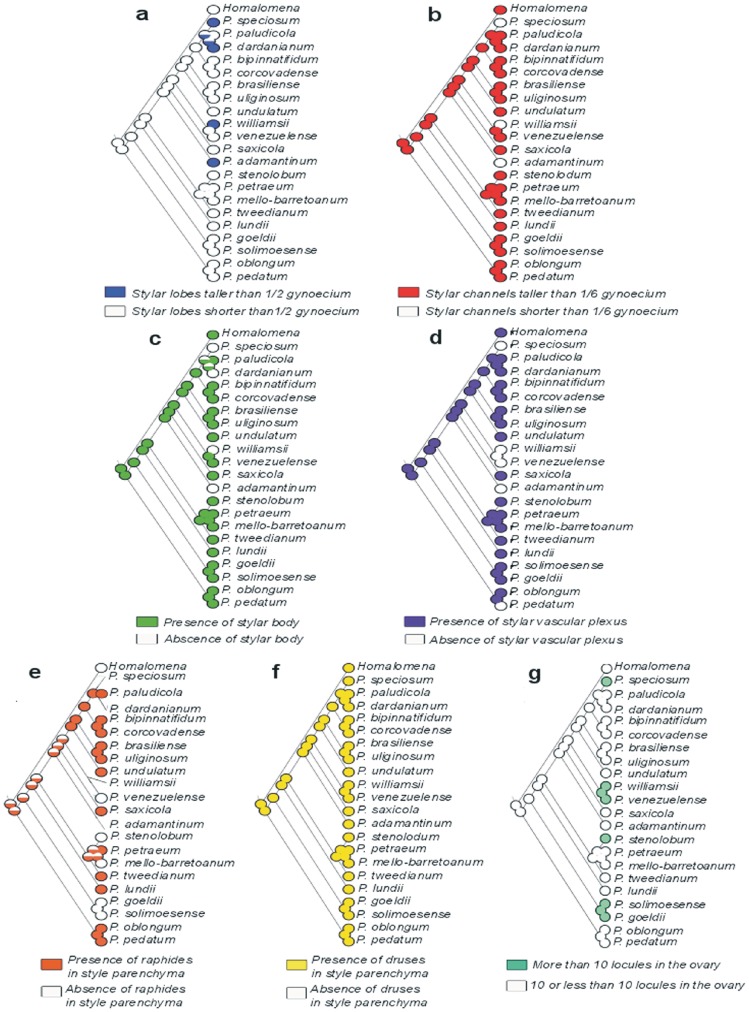
Ancestral character reconstruction of seven morphological features of gynoecium. a. Stylar lobes size, b. Stylar canal size, c. Presence of stylar body, d. Presence of stylar vascular plexus, e. Presence of raphides in the stylar parenchyma, f. Presence of druses in style and g. Number of locules in the ovary. The colored area represents the proportional likelihood of character presence.

Ancestral character state reconstruction indicated that the common ancestor of *Meconostigma* species probably possessed short stylar lobes, long stylar canals, a stylar body, a vascular plexus in the gynoecium and druses in the stylar parenchyma (Figure 6*a–f*). It is uncertain if raphide inclusions were present in the stylar parenchyma. Also, the ancestral lineage probably possessed up to 10 locules in the ovary (Figure 6*g–h*).

The topologies shown in Figure 6 suggest that the floral characters studied here are evolving independently along some branches of the tree. Specifically, two changes probably occured during the evolution of subgen. *Meconostigma* in *P. speciosum*, *P. dardanianum*, *P. williamsii* and *P. adamantinum*: an increase in height of the stylar lobes and loss of the stylar body in the gynoecium (Figure 6*a,c*). *Philodendron speciosum* and *P. williamsii* also share two independent similarities, namely, the loss of a vascular plexus (also absent in *P. venezuelense*) and the shortening of the stylar canals, which is also observed in *P. adamantinum* (Figure 6*b,d*).

The pattern of evolution of raphides in the stylar parenchyma remains unclear. From our analyses, it is not possible to affirm whether the Amazonian species (*P. venezuelense*, *P. solimoesense* and *P. goeldii*) and *P. stenolobum* have maintained the ancestral state, because the estimated likelihood of this character in the *Meconostigma* ancestral node was 50%. However, the presence of druse inclusions was inherited from the ancestral *Meconostigma* and is maintained in all species.

Finally, the Amazonian species (*P. solimoensense*, *P. venezuelense* and *P. goeldii*) and *P. speciosum*, *P. stenolobum* and *P. williamsii* show an increase in locule number in the ovary in comparison with ancestral *Meconostigma*, that probably had an ovary with less than 10 locules.

## Discussion

### Evolutionary Relationships of Subgen. *Meconostigma*

This is the first study to estimate the detailed phylogenetic relationships of *Meconostigma* with a complete sampling of extant species, and the first to use a well-established phylogeny to suggest the possible evolutionary scenario under which floral structures evolved in this group. In general, the evolutionary affinities among *Meconostigma* species were recovered with high statistical support and posterior probability. The Amazonian species *P. solimoesense* and *P. goeldii* were recovered as the sister group of the remaining *Meconostigma* species. *Philodendron solimoesense* was also recovered as basal by Gauthier *et al.* [11]. This result is, however, in sharp contrast to the phylogeny of Mayo [24], who found *P. saxicola* as the stem species of the subgenus based on data from the ovary, stigmatic region and style.

Despite the low genetic distances between the Clade 2 species pairs *P. paludicola* and *P. dardanianum*, *P. brasiliense* and *P. uliginosum*, *P. williamsii* and *P. venezuelense*, *P. petraeum* and *P. mello-barretoanum*, they are taxonomically well delimited. Thanks to the taxonomic revisions of Mayo [8] and Gonçalves and Salviani [12], subgenus *Meconostigma* is a well delimited taxon, which is favored by the small size of the group and its highly distinctive morphology. These factors lead us to believe that although these species are separated by low genetic distances, they are in fact different species.

### Gynoecial Evolution of the Species of Subgenus *Meconostigma*

We consider that estimating the divergence time of *Meconostigma* species is pivotal to elucidate the lineages diversification pattern and to understand the gynoecial evolution of the morphological characters based on their ancestry. However, the dating of *Meconostigma* evolutionary history still presents some obstacles. The only fossil assigned to the *Meconostigma* subgenus so far was described by Dilcher and Daghlian [25], based on fossilized leaves. According to the authors, the fossil would date from the Eocene of Tennessee (56,0–33,9 millions of years). However, Mayo [8] suggested that this fossil would correspond to a Peltandreae fossil, being a member of another monocot family. As there is no convergence about the taxonomy of the referred fossil and until present there is no evidence of the occurrence of *Meconostigma*, not even *Philodendron* in North America, we have decided not to use that fossil as a calibration point.

Nevertheless, as the genetic divergence is the product of the substitution rate and the time elapsed [26], an alternative way to estimate the divergence time would be to separate those components and to use the substitution rate. In *Meconostigma*, however, this approach presents two problems: (1) the substitution rates for ETS and *matK* markers have not been inferred for the Araceae family. Therefore, we would have to rely on rates estimates for other plant families; as those rates might range from orders of magnitude according to the taxon being considered, it would be very speculative to assume the substitution rates of these markers inferred for other families; (2) based on the available data, we are not able to calculate the specific substitution rate for *Meconostigma* or *Philodendron*, because this estimative depends on some calibration information, which is absent in the studied group. Therefore, our discussion about the gynoecial evolution of *Meconostigma* species will not consider the timing of the characters evolution.

Considering the morphological characters analysed in this study, except for the length of the stylar lobes of the ancestral *Meconostigma*, other floral characters differ from those observed in extant *Meconostigma* species, indicating a trend towards the maintenance of the length of the stylar lobes, the shortening of the stylar canals and the loss of vascular plexus during the evolutionary history of this group.

Our findings point to an interesting scenario underlying the evolution of these structures. Currently, it is widely accepted that the gynoecium in angiosperms corresponds to a modified form of the carpel leaf of gymnosperms [27] and that flower structures such as petals and stamens similarly correspond to modified leaves [27], [28]. In the Araceae, it is notable that flower structures tend towards naked and fused states [29], so that we observe a rather peculiar flower morphology. Our data indicate that species that present a less developed style, in other words, shorter style lobes, do not have a vascular plexus. Considering the plexus as a structure that favours the distribution of water and nutrients to the gynoecium, it would be more advantageous in flowers with more elaborate styles. On the contrary, for flowers with shorter style lobes it would be energy saving not to maintain the vascular plexus, which might be the case of *P. speciosum*, *P. williamsii*, *P. venezuelense* and *P. adamantinum* (Figure 6*c,d*).

Likewise, the longer stylar lobes and the shortening of the stylar canals over the evolutionary history of the group can be also considered advantageous to the extent that it increases the stigmatic surface, hence the likelihood that the pollen reaches the stylar canals and, consequently, the chances of fertilization. Interestingly, these features occur concomitantly in *P. speciosum*, *P. williamsii* and *P. adamantinum* (Figure 6*a,b*).

The presence of idioblasts with different calcium oxalate inclusions in the stylar body, such as druses and raphides, is commonly associated with tissue protection [30]. It is unclear if raphides were present in ancestral subgenus *Meconostigma*. Among the extant species, they are absent in *P. venezuelense*, *P. goeldii*, *P. solimoesense*, *P. mello-barretoanum* and *P. stenolobum*. It is interesting to notice that these species also have a large number of loci in the ovary (Figure 6*g*). Although it is not possible to establish a direct relationship between the number of locules and ovules, overall, the increase of locule number can be associated with the possibility of increase in ovule number. Thus, although these species potentially lack protection in the gynoecium, they are investing more energy in the chances of fertilization. An alternative hypothesis driving the increase in locule number is that this has occurred as a defense against parasitic wasp predation [31].

It is interesting that all *Meconostigma* species have druses, probably a character inherited from the common ancestor (Figure 6*f*). In view of the absence of raphide idioblasts in the gynoecium of *P. venezuelense*, *P. goeldii*, *P. solimoesense*, *P. mello-barretoanum* and *P. stenolobum*, as previously discussed, the persistence of druses might be considered advantageous in providing protection against herbivory but at a lower cost since they are less elaborate structures [30].

Under this evolutionary scenario, we propose that the morphological diversity observed in the gynoecium subgenus *Meconostigma* species is the result of an ongoing process of fusion of its floral structures. The resulting reduction of energy wastage and increase in stigmatic surface are likely to be evolving under positive selection. However, the role of natural selection and other evolutionary forces in this process still needs to be directly evaluated. Future studies addressing these issues should prove fruitful in confirming the hypotheses put forward here and, ultimately, contribute towards the understanding of inflorescence evolution in *Meconostigma* and other flowering plants.

## Supporting Information

File S1 **Supporting information that contains Table S1, Table S2, Table S3 and Table S4.** Table S1. List of GenBank accession numbers of *Philodendron* and outgroup species. Table S2. List of species, voucher information and biomes distribution. Table S3. Sequences and references of the primers used to amplify and sequence *matK* and ETS. Table S4. Morphological matrix used in the ancestral state reconstruction.
(DOC)
