# Supplementary material for: Floral Evolution of Philodendron Subgenus Meconostigma (Araceae)
Source: PLoS One. 2014 Feb 26;9(2):e89701. doi: 10.1371/journal.pone.0089701 (PMC3935929; doi:10.1371/journal.pone.0089701)
Supplement: File S1 — Supporting information that contains Table S1, Table S2, Table S3 and Table S4. Table S1. List of GenBank accession numbers of Philodendron and outgroup species. Table S2. List of species, voucher information and biomes distribution. Table S3. Sequences and references of the primers used to amplify and sequence matK and ETS. Table S4. Morphological matrix used in the ancestral state reconstruction. (DOC) [file pone.0089701.s001.doc]

**Table S1**. List of GenBank accession numbers of *Philodendron* and outgroup species. The accession numbers stated with an asterisk correspond to the ones that were not generated in this study.

| Taxon sampling | Molecular marker | |
| --- | --- | --- |
|  | 18S - ETS | *trn*K *intron- matk* |
| *Philodendron bipinnatifidum* | KF895410 | KF971323, KF981849 |
| *Philodendron pedatum* | DQ870607.1* | KF971326, KF981852 |
| *Philodendron corcovadense* | KF895417 | KF971324, KF981850 |
| *Philodendron williamsii* | KF895416 | KF971330 |
| *Philodendron venezuelense* | KF895415 | KF971329 |
| *Philodendron brasiliense* | KF895413 | - |
| *Philodendron mello-barretoanum* | KF895423 | - |
| *Philodendron dardanianum* | KF895411 | - |
| *Philodendron uliginosum* | KF895419 | - |
| *Philodendron paludicola* | KF895412 | - |
| *Philodendron speciosum* | KF895414 | - |
| *Philodendron saxicola* | KF895426 | KF971327, KF981854 |
| *Philodendron adamantinum* | KF895425 | - |
| *Philodendron petraeum* | KF895422 | KF981853 |
| *Philodendron xanadu* | KF895409 | - |
| *Philodendron undulatum* | KF895418 | KF971328, KF981855 |
| *Philodendron solimoesense* | KF895429 | KF971333, KF971333 |
| *Philodendron goeldii* | KF895428 | - |
| *Philodendron stenolobum* | KF895424 | KF971334, KF971334 |
| *Philodendron tweedianum* | KF895421 | - |
| *Philodendron leal-costae* | KF895427 | - |
| *Philodendron lundii* | KF895420 | KF971332, KF971332 |
| *Philodendron oblongum* | - | KF971325, KF981851 |
| *Homalomena cochinchinensis* | DQ870560.1* | KF971331, KF981856 |

**Table S2.** List of species, voucher information and biomes distribution. The acronyms JB refers to the botanical gardens where cultivated specimens were collected. The geographic information about these specimens are: JB Inhotim: Brumadinho / Minas Gerais; JB Plantarum: Nova Odessa / São Paulo; Bichop Museum: Hawaii; JB Rio de Janeiro: Rio de Janeiro / Rio de Janeiro.

| **Species** | **Voucher – Herbarium** | **Source – Institution** | **Biomes** |
| --- | --- | --- | --- |
| *Homalomena cochinchinensis* Engler | Calazans 36 *et al.* - RB | Cultivated – JB Inhotim | Asian Tropical Forest |
| Subgen. *Meconostigma*  *Philodendron adamantinum* Mart. ex Schott | Bastos 20 *et al.* - RFA | Cultivated – JB Plantarum | Cerrado |
| *Philodendron bipinnatifidum* Schott ex Endlicher | Calazans 4 - RB | Cultivated – JB Rio de Janeiro | Cerrado, Atlantic Forest |
| *Philodendron brasiliense* Engler | Bastos 21 *et al.* - RFA | Cultivated – JB Plantarum | Cerrado, Atlantic Forest |
| *Philodendron corcovadense* Kunth | Calazans 17 *et al.* - RFA | Field work – Maricá / Rio de Janeiro | Atlantic Forest |
| *Philodendron dardanianum* Mayo | Calazans 22 *et al.* - RB | Cultivated – JB Inhotim | Cerrado |
| *Philodendron goeldii* G.M. Barroso | Calazans 72 *et al.* - RB | Field work – Manaus / Amazonas | Amazonia |
| *Philodendron leal-costae* Mayo & G.M. Barroso | Calazans 47 - HUEFS | Field work – Milagres / Bahia | Atlantic Forest |
| *Philodendron lundii* Warm. | Calazans 41 *et al.* - RB | Cultivated – JB Inhotim | Cerrado |
| *Philodendron mello-barretoanum* Burle-Marx ex G.M. Barroso | Morais 51 - R | Cultivated – JB Rio de Janeiro | Cerrado |
| *Philodendron paludicola* E.G. Gonç. & Salviani | Calazans 38 *et al.* - RB | Cultivated – JB Inhotim | Atlantic Forest |
| *Philodendron petraeum* Chodat & Vischer | Calazans 28 *et al.* - RB | Cultivated – JB Inhotim | Cerrado |
| *Philodendron saxicola* K. Krause | Calazans 50 & Morais - RFA | Field work – Lençóis / Bahia | Cerrado |
| *Philodendron solimoesense* A.C. Sm. | Oliveira *et al.* 57 - INPA | Field work – Manaus / Amazonas | Amazonia |
| *Philodendron speciosum* Schott ex Endlicher | Morais 38 - RFA | Field work – Niterói / Rio de Janeiro | Atlantic Forest |
| *Philodendron stenolobum* E.G. Gonç. | Bastos 22 *et al.* - RFA | Cultivated – JB Plantarum | Atlantic Forest |
| *Philodendron tweedianum* Schott | Calazans 37 *et al.* - RB | Cultivated – JB Inhotim | Cerrado |
| *Philodendron uliginosum* Mayo | Calazans 19 *et al.* - RB | Cultivated – JB Inhotim | Cerrado |
| *Philodendron undulatum* Engler | Calazans 7 - RB | Cultivated – JB Rio de Janeiro | Cerrado, Atlantic Forest |
| *Philodendron venezuelense* G.S. Bunting | Calazans 26 - RB | Cultivated – JB Inhotim | Amazonia |
| *Philodendron williamsii* J.D. Hooker. | Calazans 54 & Morais - HUEFS | Field work – Itacaré / Bahia | Atlantic Forest |
| *Philodendron xanadu* Croat *et al.* | L. Mayano s/n | Cultivated – Bishop Museum | Unkown |
| Subgen. *Philodendron*  *Philodendron pedatum* (Hook.) Kunth | Morais 47 - RFA | Field work – Niterói / Rio de Janeiro | Amazonia, Caatinga, Cerrado, Atlantic Forest |
| Subgen. *Pteromischum*  *Philodendron oblongum* (Vell.) Kunth | Morais 48 - RFA | Field work – Niterói / Rio de Janeiro | Atlantic Forest |

**Table S3**. Sequences and references of the primers used to amplify and sequence *matK* and ETS.

| **Molecular marker** | **Primer sequence (5’ - 3’)** | **Reference** |
| --- | --- | --- |
| *trnK - matK* | trnK-F: GGGTTGCTAACTCAATGGTAGAG  trnK-R1: GAACCCGGA ACTHGTCGGAT | Wicke and Quandt [1] |
| 18S - ETS | ETS-AF: GACCGTGACGGYACGTGAG  18S-R: AGACAAGCATATGACTACTGGCAGG | Gauthier *et al.* [11] |

**Table S4.** Morphological matrix used in the ancestral state reconstruction. A: stylar lobes size (0: shorter than 1/2 gynoecium; 1: taller than 1/2 gynoecium); B: stylar canal size (0: channels shorter than 1/6 gynoecium; 1: taller than 1/6 gynoecium); C: stylar body (0: absent; 1: present); D: stylar vascular plexus (0: absent; 1: present); E: raphides in style (0: absent; 1: present); F: druses in style (0: absent; 1: present); G: locule number in ovary (0: 10 or less; 1: more than 10).

| **Species** | **Morphological Character** | | | | | | |
| --- | --- | --- | --- | --- | --- | --- | --- |
|  | **A** | **B** | **C** | **D** | **E** | **F** | **G** |
| *H. cochinchinensis* | - | 1 | 1 | 1 | 0 | 1 | 0 |
| *P. adamantinum* | 1 | 0 | 0 | 0 | 0 | 1 | 0 |
| *P. bipinnatifidum* | 0 | 1 | 1 | 1 | 1 | 1 | 0 |
| *P. brasiliense* | 0 | 1 | 1 | 1 | 1 | 1 | 0 |
| *P. corcovadense* | 0 | 1 | 1 | 1 | 1 | 1 | 0 |
| *P. dardanianum* | 0 | 1 | 0 | 1 | 1 | 1 | 0 |
| *P. goeldii* | 0 | 1 | 1 | 1 | 0 | 1 | 1 |
| *P. lundii* | 0 | 1 | 1 | 1 | 1 | 1 | 0 |
| *P. mello-barretoanum* | 0 | 1 | 1 | 1 | 0 | 1 | 0 |
| *P. paludicola* | 0 | 1 | 1 | 1 | 1 | 1 | 0 |
| *P. petraeum* | 0 | 1 | 1 | 1 | 1 | 1 | 0 |
| *P. saxicola* | 0 | 1 | 1 | 1 | 1 | 1 | 0 |
| *P. solimoesense* | 0 | 1 | 1 | 1 | 0 | 1 | 1 |
| *P. speciosum* | 1 | 0 | 0 | 0 | 0 | 1 | 1 |
| *P. stenolobum* | 0 | 1 | 1 | 1 | 0 | 1 | 1 |
| *P. tweedianum* | 0 | 1 | 1 | 1 | 1 | 1 | 0 |
| *P. uliginosum* | 0 | 1 | 1 | 1 | 1 | 1 | 0 |
| *P. undulatum* | 0 | 1 | 1 | 1 | 1 | 1 | 0 |
| *P. venezuelense* | 0 | 1 | 1 | 0 | 0 | 1 | 1 |
| *P. williamsii* | 1 | 0 | 0 | 0 | 0 | 1 | 1 |
| *P. pedatum* | - | 1 | ? | 0 | 1 | ? | 0 |
| *P. oblongum* | - | 1 | 1 | 1 | 1 | 1 | 0 |

**References**

1. Wicke S, Quandt D (2009) Universal primers for the amplification of the plastid trnK/matK region in land plants. An Jard Bot Madr 66(2): 285-288.
